# Proteomic Profiling of Extracellular Vesicles Derived from Cerebrospinal Fluid of Alzheimer’s Disease Patients: A Pilot Study

**DOI:** 10.3390/cells9091959

**Published:** 2020-08-25

**Authors:** Satoshi Muraoka, Mark P. Jedrychowski, Kiran Yanamandra, Seiko Ikezu, Steven P. Gygi, Tsuneya Ikezu

**Affiliations:** 1 Department of Pharmacology and Experimental Therapeutics, Boston University School of Medicine, Boston, MA 02118, USA; smuraoka@bu.edu (S.M.); sikezu@bu.edu (S.I.); 2 Department of Cell Biology, Harvard Medical School, Boston, MA 02115, USA; Mark_Jedrychowski@hms.harvard.edu (M.P.J.); steven_gygi@hms.harvard.edu (S.P.G.); 3 Abbvie Inc. Foundational Neuroscience Center, Cambridge, MA 02139, USA; kiran.yanamandra@abbvie.com; 4 Department of Neurology, Boston University School of Medicine, Boston, MA 02118, USA; 5 Center for Systems Neuroscience, Boston University, Boston, MA 02215, USA

**Keywords:** Alzheimer’s disease, biomarker, cerebrospinal fluid, extracellular vesicles, proteome

## Abstract

Pathological hallmarks of Alzheimer’s disease (AD) are deposits of amyloid beta (Aβ) and hyper-phosphorylated tau aggregates in brain plaques. Recent studies have highlighted the importance of Aβ and tau-containing extracellular vesicles (EVs) in AD. We therefore examined EVs separated from cerebrospinal fluid (CSF) of AD, mild cognitive impairment (MCI), and control (CTRL) patient samples to profile the protein composition of CSF EV. EV fractions were separated from AD (*n* = 13), MCI (*n* = 10), and CTRL (*n* = 10) CSF samples using MagCapture Exosome Isolation kit. The CSF-derived EV proteins were identified and quantified by label-free and tandem mass tag (TMT)-labeled mass spectrometry. Label-free proteomics analysis identified 2546 proteins that were significantly enriched for extracellular exosome ontology by Gene Ontology analysis. Canonical Pathway Analysis revealed glia-related signaling. Quantitative proteomics analysis, moreover, showed that EVs expressed 1284 unique proteins in AD, MCI and CTRL groups. Statistical analysis identified three proteins—HSPA1A, NPEPPS, and PTGFRN—involved in AD progression. In addition, the PTGFRN showed a moderate correlation with amyloid plaque (rho = 0.404, *p* = 0.027) and tangle scores (rho = 0.500, *p* = 0.005) in AD, MCI and CTRL. Based on the CSF EV proteomics, these data indicate that three proteins, HSPA1A, NPEPPS and PTGFRN, may be used to monitor the progression of MCI to AD.

## 1. Introduction

Alzheimer’s disease (AD) is a progressive neurodegenerative disorder and the most commonly described dementia, characterized by the accumulation of amyloid plaques in neurons [1]. Amyloid plaques are primarily composed of insoluble amyloid beta peptide (Aβ) deposits, and they represent toxicity to surrounding brain cells. Aβ deposition is most prominent in the frontal, anterior/posterior cingulate, lateral parietal, and lateral temporal regions of the brain [2,3]. In addition, neurofibrillary tangles (NFT) in neurons are also a hallmark pathology of AD and primarily consist of aggregated and hyper-phosphorylated tau protein [4]. Tau pathology, as classified by Braak and Braak, occurs in six histopathological stages [5]. In stages I and II, NFTs appear in the entorhinal cortex and hippocampus, while in stages III and IV, higher densities extend beyond the entorhinal cortex and hippocampus to the neocortex. In the final V and VI stages, pathological tau deposits are present throughout the hippocampus [5,6,7]. As AD progresses, Aβ and tau aggregates spread throughout the brain in a spatiotemporal manner [6,7].

Previously, numerous experiments showed that Aβ42 levels decreased in Cerebrospinal fluid (CSF) of patients with AD, and that the tau and p-tau levels increased compared with matched controls [8,9,10,11,12,13,14]. Recently, it has been reported that CSF Aβ42, pTau/Aβ42 and t-tau/Aβ42 were highly correlated with Aβ levels via PET imaging [15]. There are several other emerging CSF biomarkers of neuronal/synaptic injury including visinin-like protein 1, synaptosomal-associated protein 25, neurogranin, synaptotagmin-1, presynaptic marker growth-associated protein 43, and neuroinflammation-related Chitinase-3-like protein1. These markers are increased in AD and MCI compared with controls [16,17,18,19]. It was reported that proteins with high expression in the brain tissue are 1.8x enriched in CSF compared to plasma [20]. The CSF, therefore, is an attractive source to discover new biomarkers for the diagnosis and monitoring of AD progression.

Extracellular vesicles (EVs) are small lipid bilayer particles ubiquitously released by almost every cell type and present in body fluids, including urea, blood, and CSF [21,22]. EVs are classified into exosomes, which are secreted into the extracellular space after fusion of multivesicular bodies (MVBs) with plasma membrane [23] and microvesicles, which are created by the budding of the plasma membrane and release to the extracellular space [24], and apoptotic bodies, which are formed during the execution phase of the apoptotic process of living cells and released to the extracellular space [25]. EVs carry nucleic acids, such as microRNA, mRNA, and ncRNA; lipids; and proteins, all of which can be transferred to recipient cells via cell-to-cell communication [26]. EVs are being studied as potential biomarkers for cancer and neurodegeneration regions [27,28,29,30]. Recently, there have been expanded studies of the physiological and pathological functions of EVs in neurodegenerative disorders, including AD, Huntington’s disease, Parkinson’s disease, chronic traumatic encephalopathy, and Amyotrophic lateral sclerosis [31,32,33]. Furthermore, it is now known that brain-derived EVs contain pathogenic proteins, such as tau, Aβ, α-synuclein, and superoxide dismutase, and it was reported that EVs play a role in cell-to-cell propagation of the disease in brain [34,35,36,37,38,39]. It has been, moreover, reported that the EVs contain total tau (t-tau) and phosphorylated tau (p-tau), which are transported from brain to CSF in AD patients, but there are no significant differences between AD and non-demented control (CTRL) samples [40].

Known EV separation methods include differential centrifugation–ultracentrifugation, sucrose gradient ultracentrifugation, size exclusion chromatography, ultra-filtration, microfluidics, high-resolution flow cytometric sorting, polymer-based precipitation, immunoaffinity capture, affinity capture, and asymmetric-flow field-flow fractionation [41,42,43,44,45,46,47]. We have also separated the EV from CSF by ultracentrifugation, size exclusion chromatography, and MagCapture methods, and the MagCapture method provided the most enrichment of EV proteins and protein yields compatible to the LC-MS/MS/MS. Here, we provide a quantitative proteomic profiling of EVs separated from AD, mild cognitive impairment (MCI), and CTRL cerebrospinal fluid (CSF) samples by affinity capture methods and show CSF EV molecules altered during progression to AD from MCI or HC.

## 2. Materials and Methods

### 2.1. Sample Selection

CSF samples were obtained from the Banner Sun Health Research Institute (ten Alzheimer’s disease, ten Mild Cognitive Impairment, and ten control samples) [48] and the Greater Los Angeles Veteran’s Affairs Hospital (three Alzheimer’s disease) as a part of NIH NeuroBioBank. The samples from Banner Sun Health Research Institute were matched for age and sex (Table 1 and Supplementary Table S1 (Supplementary Materials)). Total plaque and tangle score for each sample in five brain regions, including entorhinal, hippocampus, frontal, parietal and temporal cortex, was obtained by estimating the density of all plaque types including compact, neuritic, classical and diffuse revealed by Thioflavin-S stains. Plaque densities were evaluated using the Consortium to Establish a Registry for Alzheimer’s Disease (CERAD) templates as none, sparse, moderate and frequent and reported numerically as 0, 1, 2 and 3, respectively [49,50]. These numbers are summed to give a score of 15 for each brain. The Institutional Review Board at Boston University School of Medicine, the Banner Health Institute and Greater Los Angeles Veteran’s Affairs Hospital approved the protocol, and all participants provided informed consent.

### 2.2. Separation of EVs from Human CSF Samples

CSF samples were centrifuged at 1200× *g* for 20 min at 4 °C, and then the supernatant was centrifuged at 10,000× *g* for 30 min at 4 °C. The subsequent supernatant was then filtered in a 0.22 μm Spin-X centrifuge tube (#CLS8160 Corning, Corning, NY, USA), and then the EV fraction was separated from the flow-through using the MagCapture Exosome Isolation Kit PS (#293-77601 Fujifilm WAKO Pure Chemical Corporation, Tokyo, Japan), according to the manufacturer’s instructions. The separated EVs were filtered in a 0.45 μm Spin-X centrifuge tube (#CLS8162 Corning) to completely remove magnetic beads.

### 2.3. Nanoparticle Tracking Analysis (NTA)

All samples were diluted in double-filtered PBS (dfPBS) at least 1:10 to get particles within the target reading range of 10–100 particles per frame on the Nanosight 300 (Malvern Panalytical Inc, Malvern, UK). Using a manual injection system, five 30 s videos were taken for each sample at 21 °C. Analysis of particle counts was carried out using Nanosight NTA 3.2 software (Malvern Panalytical Inc, Malvern, UK) with a detection threshold of 5.

### 2.4. Transmission Electron Microscopy (TEM)

The EVs separated from AD, MCI and CTRL CSF were analyzed by TEM. A total of 5µL of EV sample was adsorbed for 1 min to a carbon-coated mesh grid (#CF400-CU EMS www.emsdiasum.com) that had been made hydrophilic by 20 s exposure to a glow discharge (25 mA). Excess liquid was removed with filter paper (#1 Whatman, Little Chalfont, UK); the grid was then floated briefly on a drop of water (to wash away phosphate or salt), blotted on filter paper, and then stained with 0.75% uranyl formate (#22451 Electron Microscopy Sciences, Hatfield, PA, USA) for 30 s. After removing the excess uranyl formate using filter paper, the grids were examined, and random fields were photographed using a JEOL 1200EX TEM with an AMT 2k CCD camera.

### 2.5. Protein Concentrations

Bicinchoninic acid (BCA) assay (#23225 Pierce, Waltham, MA USA) was used to determine protein concentration for each sample. Due to the limited amount of sample, CSF was diluted 1:100 or EVs were diluted 1:10 before loading into the assay, and a 1:8 ratio of sample to reaction components was used. All assays were incubated at 60 °C for 30 min before protein concentration was read in a Biotek Synergy Mx plate reader at 562 nm.

### 2.6. In-Solution Digestion

Lysis buffer (25 mM TCEP, 5% SDS, 250 mM NaCl, Protease and Phosphatase inhibitor Cocktail (#78440 Thermo Fisher Scientific, Waltham, MA, USA) in 250 mM Na-HEPES, pH 8.8) was added to the separated EV fraction, then the mixed samples were vortexed for 5 min followed by spin down and reduced by dithiothreitol for 30 min at 60 °C. Samples were alkylated by adding 15 mM Na iodoacetamide (#I1149 Sigma-Aldrich, St. Louis, MO, USA) in the darkness for 45 min at room temperature. Subsequently, ice-cold 100% (*w*/*v*) trichloroacetic acid (TCA) (#T6399 Sigma-Aldrich) was added to samples at a final concentration of 20% TCA, then the mixed sample was incubated overnight at −20 °C and centrifuged at 15,000× *g* for 5 min in a cold room. The pellet was then washed twice with ice-cold MeOH. After lyophilization by vacuum centrifugation, the pellet was resuspended in 4 M Urea in 100 mM HEPES (pH8.5) and vortexed for 5 min. The samples were digested with proteomic grade Lys-C (#121-05063 Fujifilm WAKO Pure Chemical Corporation) in 25 mM HEPES for 2 h at room temperature with vortexing. The digested peptide was diluted with water up to 2 M Urea and digested with proteomic grade Lys-C and sequencing-grade modified trypsin (#V5111 Promega, Madison, WI, USA) in 25 mM Na HEPES overnight at 37 °C. The digested peptides were desalted by StageTip (#SP201, Thermo Fisher Scientific), and dried by vacuum centrifugation.

### 2.7. Mass Spectrometry

#### 2.7.1. Peptide Labeling with TMT 10-Plex Isobaric Labeling Kit

Tandem mass tag (TMT) labeling was performed according to the manufacturer’s instructions (#90409 Thermo Fisher Scientific). In brief, 4μL of TMT Label reagent (20 ng/μL) was added to the digested peptides in 30 μL of 200 mM HEPPS (4-(2-Hydroxyethyl)-1-piperazinepropanesulfonic acid), pH8.0. After incubation at room temperature for 1 h, the reaction was quenched with 2 μL of 5% hydroxylamine in water for 15 min. The TMT-labeled peptide samples were pooled at a 1:1 ratio across ten samples. The combined sample (36 μL) was added to 100 μL of 20% formic acid, 2 mL of 1% formic acid, desalted via StageTip, dried by vacuum centrifugation, and resuspended in 20 μL of 5% acetonitrile and 5% formic acid for nano liquid chromatography and tandem mass-spectrometry (Nano LC-MS/MS/MS).

#### 2.7.2. Nano-Liquid Chromatography and Tandem Mass-Spectrometry

Nano LC–MS/MS/MS analysis was conducted using an LTQ-Orbitrap Fusion Lumos mass spectrometer (Thermo Fisher Scientific, San Jose, CA, USA) equipped with a Proxeon EASY-nano LC 1200 liquid chromatography pump (Thermo Fisher Scientific). Peptides were separated on a 100 μm inner diameter microcapillary column packed with 35-cm long Accucore150 resin (2.6 μm, 150 Å, Thermo Fisher Scientific). We loaded 4 μL onto the column and separation was achieved using a 180 min gradient of 8 to 23% acetonitrile in 0.125% formic acid at a flow rate of ~550 nL/min. The analysis used an MS^3^-based TMT method, which has been shown to reduce ion interference. The scan sequence began with an MS^1^ spectrum (Orbitrap; resolution 120,000; mass range 400–1400 *m*/*z*; automatic gain control (AGC) target 5 × 10^5^; maximum injection time 100 ms). Precursors for MS^2^/MS^3^ analysis were selected using a Top10 method. MS^2^ analysis consisted of collision-induced dissociation (quadrupole ion trap; AGC 2 × 10^4^; normalized collision energy (NCE) 35; maximum injection time 150 ms). Following acquisition of each MS^2^ spectrum, we collected an MS^3^ spectrum using our recently described method in which multiple MS^2^ fragment ions were captured in the MS^3^ precursor population using isolation waveforms with multiple frequency notches [51]. MS^3^ precursors were fragmented by high-energy collision-induced dissociation (HCD) and analyzed using the Orbitrap (NCE 65; AGC 1 × 10^5^; maximum injection time 150 ms, resolution was 50,000 at 200 Th).

#### 2.7.3. Mass-Spectrometry Data Analysis

A compendium of in-house developed software was used to convert mass spectrometric data (Raw file) to the mzXML format, as well as to correct monoisotopic *m*/*z* measurements [52]. Database searching included all entries from the Homo sapiens UniProt database (version October 2018). This database was concatenated with one composed of all protein sequences in the reversed order. Searches were performed using a 50 ppm precursor ion tolerance for total protein level profiling [51]. The product ion tolerance was set to 0.9 Da, which was chosen to maximize sensitivity in conjunction with SEQUEST searches and linear discriminant analysis. TMT tags on lysine residues and peptide N termini (+229.163 Da) and carbamidomethylation of cysteine residues (+57.021 Da) were set as static modifications, while oxidation of methionine residues (+15.995 Da) was set as a variable modification. Peptide–spectrum matches (PSMs) were adjusted to a 1% false discovery rate (FDR). Filtering was performed using an in-house linear discrimination analysis (LDA) method to create one combined filter parameter from the following peptide ion and MS^2^ spectra metrics: SEQUEST parameters XCorr and ΔCn, peptide ion mass accuracy and charge state, in-solution charge of peptide, peptide length, and mis-cleavages. Linear discrimination scores were used to assign probabilities to each MS^2^ spectrum for being assigned correctly, and these probabilities were further used to filter the dataset with an MS^2^ spectra assignment FDR of less than 1% at the protein level [53]. For TMT-based reporter ion quantitation, we extracted the summed signal-to-noise (S/N) ratio for each TMT channel and found the closest matching centroid to the expected mass of the TMT reporter ion. PSMs were identified, quantified, and collapsed to a 1% peptide FDR and then collapsed further to a final protein-level FDR of 1%. Moreover, protein assembly was guided by principles of parsimony to produce the smallest set of proteins necessary to account for all observed peptides. Proteins were quantified by summing reporter ion counts across all matching PSMs. PSMs with poor quality, MS^3^ spectra with more than eight TMT reporter ion channels missing, MS^3^ spectra with TMT reporter summed signal-to-noise ratio less than 100, or no MS^3^ spectra were excluded from quantification [54]. The mass spectrometry proteomics data have been deposited to the ProteomeXchange Consortium via the PRIDE partner repository with the dataset identifier as specified in the footprint [55]. Protein quantitation values were exported for further analysis in Microsoft Excel or Prism6. Each reporter ion channel was summed across all quantified proteins.

### 2.8. Measurement of Total Tau (t-tau) and Tau Phosphorylated on Threonine 231 (p-tau231)

M-PER^®^ Mammalian Protein Extraction Reagent (#78503 Pierce) was added to the isolated EV fraction using MagCapture Exosome Isolation Kit PS with Halt™ Protease and Phosphatase Inhibitor Cocktail (#78442 Thermo Fisher Scientific) and was mixed by vortexing for 15 min. The lysed EVs were filtered by 0.45 μm Spin-X centrifuge tube (#CLS8162 Corning). The EV t-tau and p-tau231 were measured using the Simoa Tau advantage kit (#101522 Quanterix, Lexington, KY, USA) and Simoa pTau-231 Advantage kit (#102292 Quanterix) on the Simoa HD-1 analyzer (Quanterix). All CSF-derived EV samples were diluted 10x with the Tau Calibrator Diluent (#101631 Quanterix) prior to the assays, to minimize matrix effects, and were analyzed in duplicate on one occasion. The relative concentration estimates of t-tau and p-tau231 were calculated according to the standard curve.

### 2.9. Statistical Analysis

Statistical analysis was conducted using IBM SPSS software version 26 and GraphPad Prism 6. Between-group comparisons were analyzed by one-way ANOVA followed by Tukey’s HSD test for multiple comparisons. Bivariate correlation analysis examined differences between AD, MCI and CTRL in proteomics data and demographics data by Spearman’s rank using IBM SPSS. Gene Ontologies of identified proteins were elucidated by DAVID Bioinformatics Resources version 6.8 (https://david.ncifcrf.gov). Protein networks and pathway analysis were generated using Ingenuity Pathway Analysis (IPA). Venn diagram analyses were generated using Venny 2.1 (http://bioinfogp.cnb.csic.es/tools/venny/).

## 3. Results

### 3.1. Workflow for Protein Profiling of Extracellular Vesicles Separated from Human CSF

**Figure 1:**
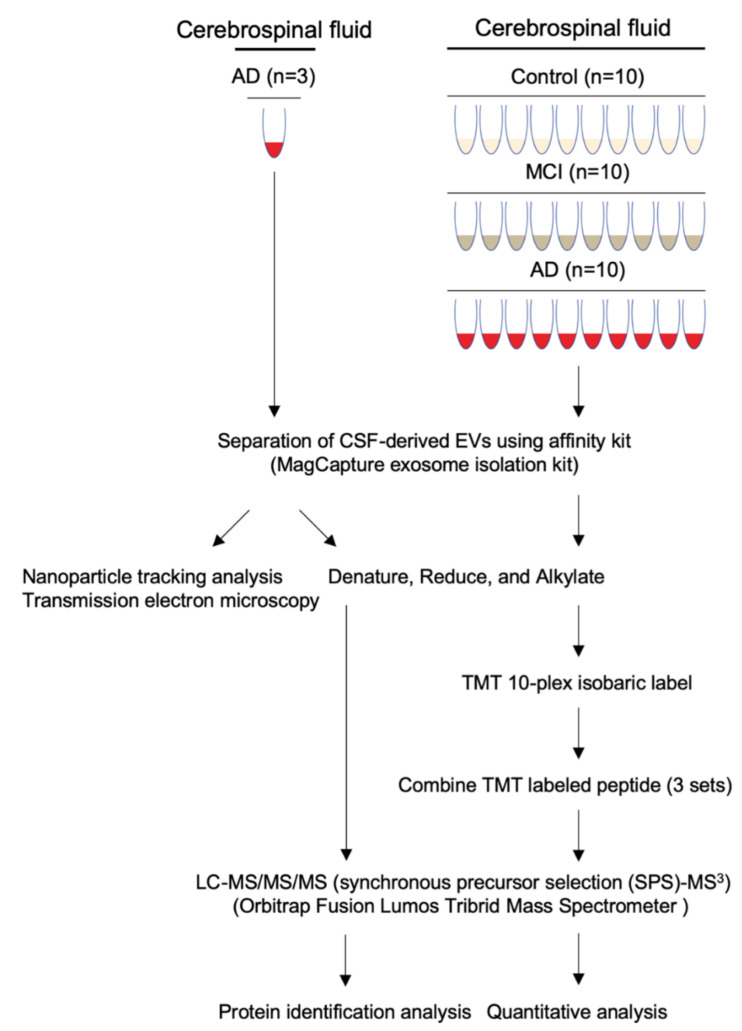
Workflow used in proteomics analysis of CSF-derived EVs: EVs were separated from control, MCI, and AD CSF using the Affinity Capture method (MagCapture Exosome Isolation kit). The separated EVs were denatured, reduced and alkylated, followed by Lys-C and trypsin digestion, and labeled with a TMT 10-plex isobaric label kit for quantitative proteomics analysis. The non-labeled peptide (**left**) and combined TMT-labeled peptide (**right**) were analyzed by MS^3^ on an Orbitrap Fusion Lumos Tribrid Mass Spectrometer.

Our experimental design for protein profiling of CSF-derived EVs is outlined in Figure 1. The EVs were separated from human AD (*n* = 13), MCI (*n* = 10) and CTRL (*n* = 10) CSF by the Affinity capture method (MagCapture Exosome isolation kit). Subsequently, EVs separated from pooled CSF with AD (*n* = 3) were examined by particle number and size using Nanoparticle tracking analysis (NTA) and by morphology using Transmission electron microscopy (TEM). Protein profiling was done using label-free Mass spectrometry. EVs separated from AD (*n* = 10), MCI (*n* = 10) and CTRL (*n* = 10) CSF were identified and quantified by TMT-labeled Mass spectrometry for EV-based monitoring of AD progression.

### 3.2. Biochemical and Morphological Characterization of EVs Separated from CSF

**Figure 2:**
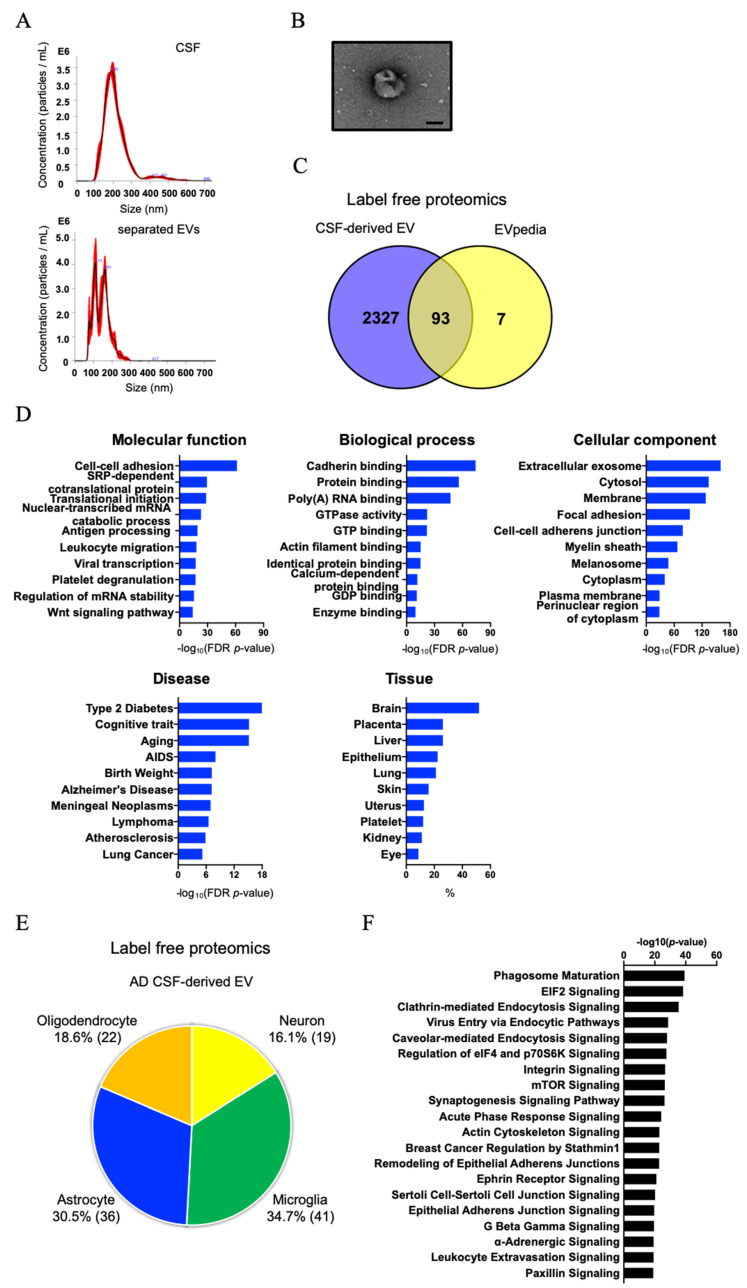
Characterization of EVs separated from human CSF by Affinity Capture: (**A**) EVs separated from CSF were examined by size and number using Nanoparticle Tracking Analysis. The black line shows the curve fitting; the red line represents the error of mean of the quadruplicate measurements. Y axis: EV particle counts (/mL), X axis: EV particle size [nm]. Upper panel: CSF, Lower panel: separated EV fraction. (**B**) TEM image of EVs separated from CSF. Scale bar = 100 nm. (**C**) Venn diagram of the proteins identified in CSF-derived EVs by label-free proteomics analysis and EVpedia Top100. (**D**) Gene Ontology (GO) analysis using DAVID Bioinformatics Resources 6.8. The GO terms of Top10 results for ‘Molecular function’, ‘Biological process’, ‘Cellular Component’, ‘Disease’ and ‘Tissue’ with −log_10_ (FDR *p*-value) or %. (**E**) Enrichment of brain cell-specific markers in CSF-derived EV proteins and brain-derived EV proteins. Yellow: Neuron, Green: Microglia, Blue: Astrocytes, Orange: Oligodendrocytes. The parentheses show the percentage of identified cell type-specific proteins. (**F**) Canonical pathways of AD CSF-derived EV proteins in the IPA analysis.

The peak diameter was 192 nm for CSF samples and 111 and 161 nm for EV fraction (Figure 2A and Table 2). The EVs separated from CSF using the MagCapture exosome isolation kit contained the two subtypes [56]. The particle counts were 4.60 × 10^9^ particles in 500 μL of CSF and 2.36 × 10^9^ particles in 100 μL in separated EV fraction (Table 2). A significance difference in protein content was observed between CSF and separated EV fraction, and the particles per protein were 3.87 × 10^6^ (particles/μg) in CSF and 3.03 × 10^9^ (particles/μg) in separated EV fraction (Table 2). The result suggests that the affinity capture method can concentrate EV particles from CSF efficiently and remove contaminant proteins. Figure 2B shows a representative TEM image of separated EV fraction, possessing a cup-shaped morphology. Label-free Mass spectrometry analysis of the separated EV fraction identified a total of 2546 proteins (Supplementary Table S2 (Supplementary Materials)). Figure 2C shows a Venn diagram comparing identified proteins with the top100 EV proteins from the EVpedia database [57]. We found that the EV fraction was presented in tetraspanins such as CD9, CD63 and CD81, annexins, endosomal sorting complexes required for transport (ESCRT) complexes including vacuolar sorting (VPS) protein and Rab family, and non-EV molecules such as apolipoprotein, as listed in the MISEV2018 guidelines [21] (Supplementary Table S3 (Supplementary Materials)). We submitted the proteomics dataset to Gene Ontology (GO) with Database for Annotation, Visualization, and Integrated Discovery (DAVID) [58]. The identified proteins were significantly enriched for Extracellular exosomes and for cognitive traits, Aging/Telomere Length, and Alzheimer’s disease as determined by DAVID cellular component and disease ontology (Figure 2D). Interestingly, the DAVID tissue category showed that CSF-derived EVs were enriched for brain-specific proteins. Thus, we next searched for brain cell-type specific molecules within the CSF-derived EV proteomics dataset using the mouse proteomics dataset as a reference [59]. The top100 cell type-specific molecules, which have at least 2-fold change in concentration in the cell type of interest over other cell types, were screened against our EV proteomics dataset. The distribution of these markers indicates that, in the human CSF, 16.1% of the identified molecules are of neuronal origin, whereas the other 83.9% of EV proteins are of glial origin, including microglia, astrocytes, and oligodendrocytes (Figure 2E and Supplementary Table S4 (Supplementary Materials)). We submitted the identified proteins to Ingenuity pathway analysis (IPA) (Figure 2F): the EV proteins from CSF were involved in glial-related signaling in canonical pathways.

### 3.3. Comparison of AD, MCI, and CTRL CSF-Derived EV Proteins by TMT-Labeled Quantitative Proteomics Analysis

**Figure 3:**
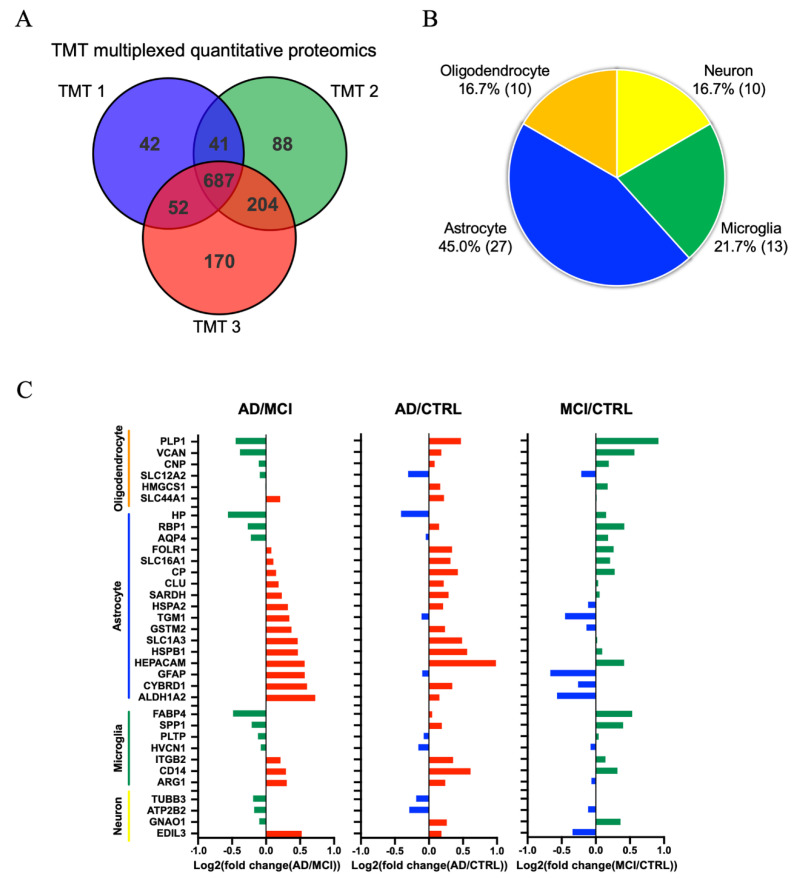
Proteomics comparison of AD, MCI and CTRL CSF-derived EV protein: (**A**) Venn diagram showing the number of proteins reproducibly identified among the three separate TMT-MS. (**B**) Enrichment of brain cell-specific markers in CSF-derived EV proteins and brain-derived EV proteins. Yellow: Neuron, Green: Microglia, Blue: Astrocytes, Orange: Oligodendrocytes. The parentheses show the number of identified cell type-specific proteins. (**C**) Comparison of the cell type-specific proteins in AD CSF-derived EVs, MCI EVs and CTRL EVs. The left panel shows AD vs. MCI. The red bar shows higher expression in AD compared with MCI and the green bar indicates higher expression in MCI compared with AD. The middle panel indicates AD vs. CTRL. The red bar shows higher expression in AD compared with CTRL and the blue bar indicates higher expression in CTRL compared with AD. The right panel shows MCI vs. CTRL. The green bar shows higher expression in MCI compared with CTRL and the blue bar indicates higher expression in CTRL compared with MCI.

To identify molecules reflecting the progression from MCI to AD, the EVs were separated from ten AD, ten MCI and ten CTRL CSF samples by the affinity capture method, and analyzed by TMT-labeling mass spectrometry. A total of 1284 proteins were identified and quantified (Figure 3A). We tested for brain cell type-specific molecules within the TMT-labeled proteomics dataset using the mouse proteomics dataset as a reference [59]. The distribution of these markers indicates that, in the human CSF, 16.7% of the identified molecules are of neuronal origin, whereas the other EV proteins are of glial origin (Figure 3B). Moreover, among the 687 proteins common to the three TMT-sets, differences in the expression of cell type-specific markers were observed (Supplementary Table S5 (Supplementary Materials) and Figure 3C). Interestingly, almost cell type-specific molecules were enriched in the AD and MCI groups (Figure 3C). Astrocyte-specific molecules were enriched in AD compared to MCI. The EV proteins identified by TMT-labeling proteomics were involved in glial-related signaling in canonical pathways. (Supplementary Figure S1 (Supplementary Materials)). We have also assessed the potential blood contamination in the CSF samples by examining the molecules enriched in red blood cells [60,61,62,63], and only hemoglobin beta (HBB) and peroxiredoxin-2 (PRDX2) were identified in the proteomic profiles of CSF EVs (Supplementary Figure S2 (Supplementary Materials)). There was no statistical significance in the amount of HBB or PRDX2 among the 3 groups and no outlier was detected, ruling out the possibility of blood contamination.

### 3.4. Molecules Altered during Progression to Alzheimer’s Disease from Mild Cognitive Impairment

**Figure 4:**
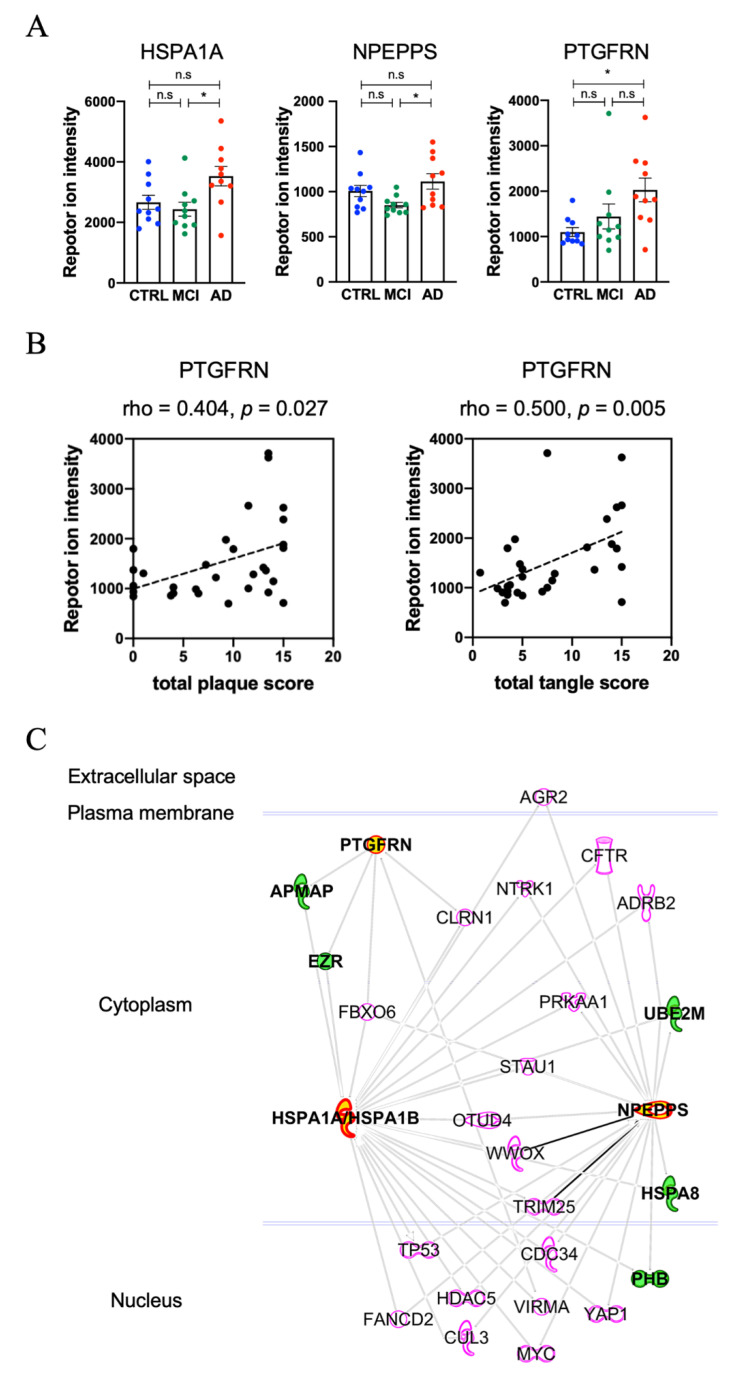
Altered EV molecules during progression from MCI to AD: (**A**) A scatter plot of TMT reporter ion intensity as measured by proteomics per selected candidate protein (HSPA1A, NPEPPS, PTGFRN). One-way ANOVA, followed by Tukey’s-HSD multiple test (n.s: non-significant, * *p* < 0.05 for AD vs. MCI or AD vs. CTRL). (**B**) A scatter plot showing correlations between demography score and PTGFRN expression level, which was measured using TMT proteomics. The spearman’s rho for total plaque was 0.404 (*p* = 0.027), and for total tangle was 0.500 (*p* = 0.005). (**C**) Protein–Protein interaction networks of three candidate proteins identified by IPA. The red symbols represent three candidate proteins (HSPA1A, NPEPPS and PTGFRN), while the green symbols represent proteins identified by TMT-labeling proteomics.

We measured t-tau and p-tau_231_ in EVs separated from different CSF cohorts. Neither t-tau nor p-tau_231_ was significantly different among the three groups (Supplementary Table S6 and Figure S3 (Supplementary Materials)). This suggests that the CSF EVs containing tau and p-tau may be unaltered between AD and MCI. Based on bioinformatic analysis, we identified candidate molecules that distinguish AD from MCI. The significantly differentially expressed proteins found in the AD and MCI or CTRL samples are listed in Table 3. Figure 4A shows the scatter plot of the three candidate proteins, Heat shock 70 kDa protein 1A (HSPA1A), Puromycin-sensitive aminopeptidase (NPEPPS), and Prostaglandin F2 receptor negative regulator (PTGFRN). These proteins were differentially expressed, among the AD, MCI and CTRL groups, to a statistically significant degree. The PTGFRN shows a significant positive association with total plaque score and total tangle score (Figure 4B), suggesting its prediction potency of AD progression. Figure 4C shows the protein–protein interaction networks of HSPA1A, NPEPPS and PTGFRN identified by IPA. This approach identified adipocyte plasma membrane-associated protein (APMAP) and ezrin (EZR) as directly interacting molecules for both PTGFRN and HSPA1A/HSPA1B, and NEDD8-conjugating enzyme Ubc12 (UBE2M), Heat shock 70 kDa protein 8 (HSPA8) and prohibitin (PHB) as directly interacting with HSPA1A/HASPA1B and NPEPPS among the identified molecules in the CSF EVs. These two macromolecular complexes may be present in the same or different EVs in the human CSF samples.

## 4. Discussion

In the present study, we separated EVs from CSF of AD, MCI and CTRL samples by the Affinity capture method, and then performed label-free and TMT-labeled quantitative proteomic profiling by Nano LC-MS/MS. We identified 2546 proteins in AD CSF-derived EVs by label-free proteomics. Next, 1284 proteins were quantified among the three groups by TMT-labeled quantitative proteomics, three proteins of which show significant differences in expression across the three groups. In CSF, EVs released from brain cells are present, and the glial cell type-specific molecules were enriched in AD and MCI compared to CTRL.

The affinity capture method employed here has been reported to have a higher purity of EV than other exosome isolation kits in downstream proteomics analysis, resulting in lower protein yields [46]. This method captures the heterogeneity of phosphatidylserine (PS) + EV, including apoptotic bodies from cell culture medium and body fluids, by binding to PS present on the membrane surface of EV. It has been reported that two subtypes of small EVs containing different rates of PS+ EV could be separated by gradient ultracentrifugation methods from culture media of cancer cell lines [56]. Furthermore, it showed the freezing/thawing cycle increase the rates of PS+ EV in the two subtypes of sEVs. It is not known how much the PS- EVs exist in biospecimens, but PS- EVs cannot be isolated by this method compared to other EV isolation kits. This may be one of the possible causes for the difference between our results and the reported CSF EV tau and p-tau [64].

The protein levels of HSPA1A and NPEPPS were significantly different in AD CSF EVs compared to MCI. HSPA1A is a major heat shock protein, which is expressed abundantly in almost all cells. It is critical for the cellular management of environmental stress by preventing abnormal tau aggregation [65,66]. It has been reported that the HSPA1A interacts with APMAP, which is expressed in neurons, and their proteins’ dysfunction was shown to increase Aβ40 and Aβ42 levels by destabilization of lysosomes [67,68]. HSPA1A gene expression was down-regulated in late stage AD compared to early stage in the prefrontal cortex [69] and its protein level was also downregulated in AD brain [68], suggesting that increased levels of HSPA1A in CSF EV may represent neuronal cell loss in the affected brain regions. NPEPPS is a cytosolic aminopeptidase and highly expressed in the central nervous system. Karsten et al. have reported that NPEPPS was upregulated in the cerebellum of human P301L tau mutant transgenic mice, and shown to protect against tau-induced neurodegeneration [70,71]. NPEPPS digests soluble tau purified from non-pathological human brain but not the tau purified from AD brain [72], suggesting its role in the clearance of normal tau in a posttranslational modification-sensitive manner. CSF NPEPPS is investigated as a biomarker candidate in AD patients [73]. Thus, the combination of HSPA1A and NPEPPS in CSF EVs may serve as a potential biomarker in monitoring the conversion of MCI to AD. PTGFRN, a member of tetraspanin family, was significantly increased in AD compared to CTRL and showed a significant positive association with total plaque score or total tangle score. PTGFRN interacts with gamma–secretase complex, and gene silencing of PTGFRN decreased Aβ40 and Aβ42 production, suggesting its critical role in the gamma secretase activity [68,74]. Therefore, the three key proteins described in this work, HSPA1A, PTGFRN and NPEPPS, may have direct implications in AD pathogenesis and serve as monitoring tools for AD progression.

In summary, among a total of 687 CSF EV common proteins among three TMT-labeled proteomic analyses, the levels of HSPA1A, NPEPPS and PTGFRN were significantly increased in AD CSF EVs compared to MCI CSF EVs. The small sample size of AD, MCI and CTRL CSF may have caused the identification of the limited number of proteins. Thus, it is necessary to replicate this TMT-labeled proteome with a larger sample size, or perform validation study using other body fluids, such as blood, to determine if these proteins serve as potential biomarkers in the progression from CTRL or MCI to AD. Recent developments in immunoassay, including ExoView, Single Molecule Array (Simoa), microfluidic chips and targeted MS, are expected to lead the three proteins to clinical diagnosis applications.

## Figures and Tables

**Table 1:** Patient information.

**For Label-Free Proteomics**	AD					
	(*n* = 3)					
**Age, mean**	82.7 ± 7.57					
Gender	2M, 1F					
**For TMT-Label Proteomics**	CTRL	MCI	AD	*t*-test *^a^* (*p*-value *^b^*)	*t*-test *^a^* (*p*-value *^b^*)	*t*-test *^a^* (*p*-value *^b^*)
	(*n* = 10)	(*n* = 10)	(*n* = 10)	(CTRL_MCI)	(CTRL_AD)	(MCI_AD)
Age, mean	89.8 ± 6.12	89.0 ± 5.27	87.1 ± 4.41	0.313 (0.758)	1.131 (0.273)	0.874 (0.393)
Gender *^c^*	5M, 5F	5M, 5F	5M, 5F			
PMI, mean	2.90 ± 0.85	2.94 ± 0.87	2.84 ± 0.49	−0.106 (0.917)	0.173 (0.865)	0.299 (0.768)
Braak stage	2.8 ± 0.42	3.3 ± 0.67	5.4 ± 0.52	−1.987 (0.062)	−12.333 (<0.001)	−7.814 (<0.001)

*^a^* The group comparisons were performed using independent t-test. *^b^* The statistical significance of the differences were calculated using a two-tailed test. *^c^* M: Male, F: Female.

**Table 2:** Enrichment of EVs separated from CSF by the Affinity capture kit.

	Human CSF	EV Fraction
Mode size (nm) *^a^*	191	110
Particle number *^a,b^*	4.6 × 10^9^/500 μL CSF	2.36 × 10^9^/100 μL
EV protein (μg) *^b,c^*	1187.48	0.78
Particles/proteins (μg)	3.87 × 10^6^	3.03 × 10^9^

*^a^* Particle number and size of isolated EVs were measured by Nanoparticle Tracking Analysis (NTA), *^b^* The starting material is mouse whole brain, *^c^* The EV proteins were measured by BCA.

**Table 3:** Up- and down- regulated EV proteins in AD or MCI compared to control group.

Protein ID	Gene Symbol	log_2_	log_2_	log_2_	One-Way ANOVA *^a^*	−log_10_ (*p*-Value	−log_10_ (*p*-Value	−log_10_ (*p*-Value
		(AD/CTRL)	(MCI/CTRL)	(AD/MCI)		(AD_CTRL)) *^a^*	(MCI_CTRL))	(AD_MCI))
P08107	HSPA1A	0.405	−0.128	0.533	0.017	1.149	0.087	1.721
Q9P2B2	PTGFRN	0.882	0.391	0.491	0.024	1.721	0.268	0.75
P55786	NPEPPS	0.145	−0.243	0.388	0.025	0.319	0.666	1.699

*^a^* The group comparisons were performed by one-way ANOVA and post hoc Tukey test.

## Supplementary Materials

The following are available online at https://www.mdpi.com/2073-4409/9/9/1959/s1. Figure S1: The canonical pathway of identified proteins by TMT-labeling quantitative proteomics in IPA. Figure S2: Quantitative analysis of HBB and PRDX2. Figure S3: Comparison of total tau and tau phosphorylated at threonine 231 in EVs among AD, MCI and CTRL. Table S1: Patient Demographic Information. Table S2: Identification of EV proteins isolated from AD CSF by label-free proteomics analysis. Table S3: Identification of the EV and non-EV marker proteins. Table S4: Cell type specific list for label-free proteomics dataset. Table S5: Identification and quantification of EV proteins separated from AD, MCI, and CSF by TMT-labeled proteomics analysis. Table S6: Sample used for t-tau and p-tau ELISA assay.
