# Supplementary material for: Proteomic Profiling of Extracellular Vesicles Derived from Cerebrospinal Fluid of Alzheimer’s Disease Patients: A Pilot Study
Source: Cells. 2020 Aug 25;9(9):1959. doi: 10.3390/cells9091959 (PMC7565882; doi:10.3390/cells9091959)
Supplement: Supplementary file 1 [file cells-09-01959-s001.zip › cells-863911-supplementary-publication/Supplementary Figure_R3.pdf]

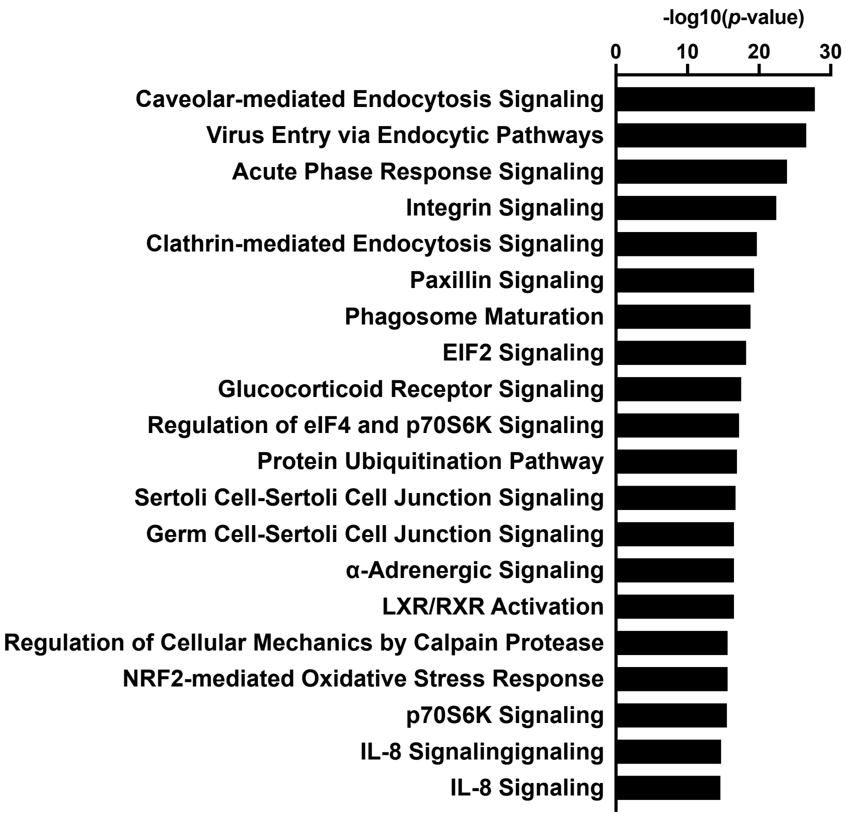

Supplementary Figure S1. The canonical pathway of identified proteins by TMT-labeling quantitative proteomics in IPA.

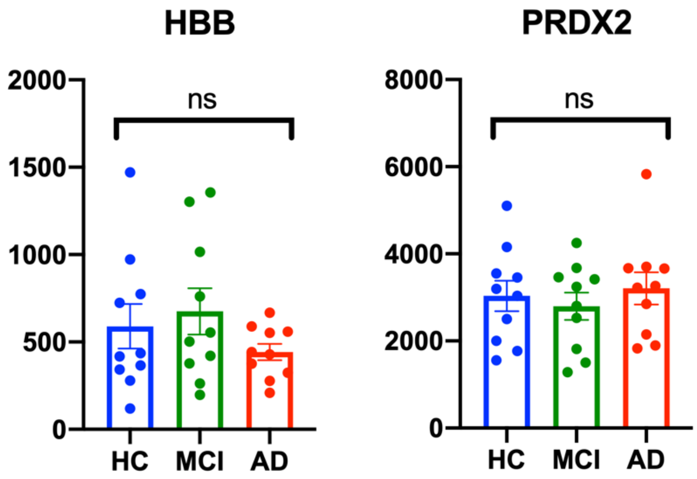

Supplementary Figure S2. Quantitative analysis of HBB and PRDX2. ns: no significance as determined by one-way ANOVA. Outlier identifier with "Robust nonlinear regression and Outlier removal method" (ROUT, Q = 1%) detected no outlier among tested samples (Prism 8, GraphPad).

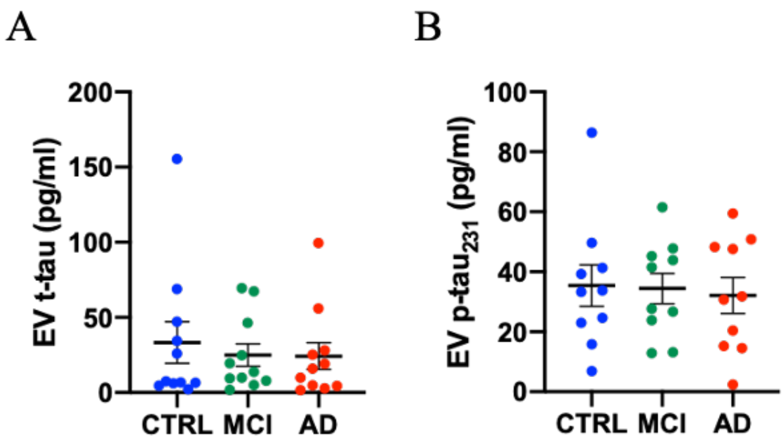

Supplementary Figure S3. Comparison of total tau and tau phosphorylated at threonine 231 in EVs among AD, MCI and CTRL: **A)** total tau; there is no significantly different. **B)** p-tau$_{231}$; there is no significant difference among groups (A, B).
